# Modulation Techniques for Biomedical Implanted Devices and Their Challenges

**DOI:** 10.3390/s120100297

**Published:** 2011-12-28

**Authors:** Mahammad A. Hannan, Saad M. Abbas, Salina A. Samad, Aini Hussain

**Affiliations:** Department of Electrical, Electronic & Systems Engineering, Faculty of Engineering and Built Environment, Universiti Kebangsaan Malaysia, 43600 UKM Bangi Selangor, Malaysia; E-Mails: saadabas@eng.ukm.my (S.M.A.); salina@eng.ukm.my (S.A.S.); aini@eng.ukm.my (A.H.)

**Keywords:** modulation techniques, bio-medical/wireless implanted devices, power amplifiers, inductive coupling link

## Abstract

Implanted medical devices are very important electronic devices because of their usefulness in monitoring and diagnosis, safety and comfort for patients. Since 1950s, remarkable efforts have been undertaken for the development of bio-medical implanted and wireless telemetry bio-devices. Issues such as design of suitable modulation methods, use of power and monitoring devices, transfer energy from external to internal parts with high efficiency and high data rates and low power consumption all play an important role in the development of implantable devices. This paper provides a comprehensive survey on various modulation and demodulation techniques such as amplitude shift keying (ASK), frequency shift keying (FSK) and phase shift keying (PSK) of the existing wireless implanted devices. The details of specifications, including carrier frequency, CMOS size, data rate, power consumption and supply, chip area and application of the various modulation schemes of the implanted devices are investigated and summarized in the tables along with the corresponding key references. Current challenges and problems of the typical modulation applications of these technologies are illustrated with a brief suggestions and discussion for the progress of implanted device research in the future. It is observed that the prime requisites for the good quality of the implanted devices and their reliability are the energy transformation, data rate, CMOS size, power consumption and operation frequency. This review will hopefully lead to increasing efforts towards the development of low powered, high efficient, high data rate and reliable implanted devices.

## 1. Introduction

Biomedical implantable devices have been available for more than sixty years. The first transistorized biomedical implanted device was designed and developed by Earl Bakken in 1957 for the cardiac pacemaker [1]. Investigations on implanted devices have been focused of the most important issues of biomedical implants, which are patient safety and comfort [2]. This can be achieved by reducing the power consumption and ensuring efficient energy transfer to the implanted devices [3]. Therefore, wireless energy transfer is an important issue for implanted devices [4].

Implanted medical devices are electronic devices that monitor and diagnose the electromyography (EMG), electrocardiogram (ECG), electroretiuogram (ERG) and electrooculography (EOG) of the patient and send current to various parts of a patient body. In general, the implanted device consists of two parts: the internal part located underneath the body skin and an external part. *i.e.*, controller [5,6]. The external part is used for powering the combination and sending data to the outside world. Implantable devices are self-operating devices which adjust their operation depending upon the patient’s condition. These devices do not rely on external sources of power. Thus, low power consumption and high data rate are the main requirements for medical implant devices [7]. In order to minimize cost, patient trauma and risk associated with the repeated surgeries, it is necessary to increase the lifetime of implanted batteries by conserving energy at every stage of a device’s operation. Thus, in order to consume the power during monitoring and diagnosis, implanted devices powered by RF signals through inductive coupling links are used to reduce the complexity of the system [8].

There are many methods to conserve power and reduce the interference on nearby electronics by choosing the suitable modulation and suitable design of the modulator and demodulator. Details of the modulation and demodulation of wireless telemetry and implanted devices such as amplitude shift keying (ASK), frequency shift keying (FSK) and phase shift keying (PSK) have been discussed in many reviews. However, still there are many drawbacks with the communication systems of the existing designed and developed devices. There are a lot of papers in the field of wireless low power electronics, such as optical biomedical sensing [9], cochlear implants [10] biomedical wireless sensor networks [11], intelligent biomedical devices [12] and body sensors [13]. This paper provides a detailed review of the literature concerning the design of low power transmitters for different forms of digital bandpass modulations used in implanted devices covering the period from 2000 to 2011. The purpose of this review is to discuss and classify all the types of digital modulation used in wireless telemetry bio-devices and biomedical implanted devices to provide a good background on the challenges and problems that are being faced and to develop appropriate solutions.

## 2. Systems Overview

**Figure 1.**
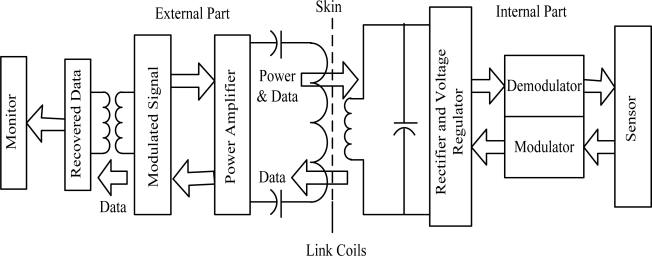
Architecture for data and power transmission system.

In general, wireless telemetry bio-devices and biomedical implanted devices mainly consist of two parts: an external one, located outside the human body and an internal part, located inside the human body. The external part is used to supply power and transmit the data to the internal part through an inductive coupling link as well as monitor the data from the human body [14,15]. Details of the data transmission between the parts are shown in Figure 1.

### 2.1. Power Supply Technologies

The power supply technologies used in the aforesaid devices can be classified according to the applications of the implanted devices. Generally, the implanted devices can be powered by batteries such as in the case of pacemakers, cochlear implants, retinal prosthesis, brain implants, *etc.* However, the limited lifetime of the implanted batteries lead to many challenges to improve their longevity. The wireless telemetry bio-devices can also be powered by radio frequency (RF) signals, transmitting power from the external part to the internal part through an inductive coupling coil and then converted into AC and DC voltage [16]. Another source of harvested energy is used to power the implanted devices such as knee implants that use the vibration of piezoelectric materials [17] or body motion [18].

### 2.2. Wireless Communication Technologies for Implanted Devices

**Figure 2.**
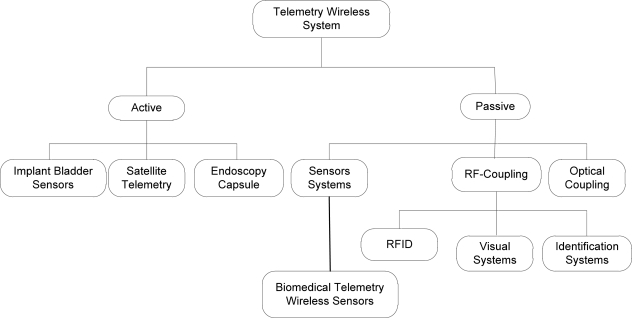
Communication technologies for wireless telemetry.

The biomedical implanted devices can communicate with the exterior world by up-link, and using inductive wireless coupling or UHF links to transmit data from the body which is saved in sensors [19]. Most bio-devices are bi-directional systems with transmitted RF signal carriers either swallowed or surgically implanted in a patient. The communication between the two parts is possible even through different media such as biological tissues like fat, muscle, blood, bone, *etc.* [20,21]. The classification types of the most common passive and active wireless communications are shown in Figure 2.

### 2.3. Characteristics of the Implantable Devices

There are several characteristics that are shared by most biomedical implantable devices as follows:

- *Low Power Consumption*: low power consumption is the main requirement for medical implant devices where the large dissipation in power increases the possibility to damage the soft tissues in the human body. Changing or charging batteries can be inconvenient, difficult, costly and even risky for the patient; all implantable medical devices need to consume as little energy as possible.
- *High Reliability:* A failure of an implantable medical device can result on inconvenience, pain, damage or even death for the patient. Maintenance is also costly and risky. Thus, reliability for the implanted devices must be very high.
- *Low Voltage Signals*: Most of the natural signals are inside the human bodies as well as the output of the transducers are in the *V* or *mV* range, which requires special care in sensing and amplifying.
- *Low Frequencies:* The natural frequency span of biological signals varies from a fraction of a hertz to several kilohertz, However, most of the implantable devices are powered by low-frequency (<1 MHz signal) magnetically coupled coils (often modulated to include the data telemetry). Nowadays, the designers design the bio-implanted devices to operate in the medical implant communications service (MICS) band.
- *Small Size:* Implantable devices need to be as small as possible, so as to be less invasive to the human body. This does not always means that the silicon area should be as small as possible, because increasing the silicon area to minimize external components can reduce overall size. In addition the use of some methods such as auto-zeroing techniques, FPGA methods, and artificial intelligence methods can help minimize the overall size.

## 3. Modulation Techniques

**Figure 3.**
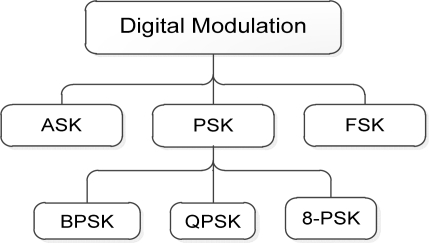
Modulation techniques used in biomedical devices.

Digital modulation techniques impress the digital signal onto a carrier signal for data transmission. A sequence of digital data is used to alter the parameter of a high frequency called carrier signal. Thus, signal transmission takes place by modulating different parameters like amplitude, phase and frequency of the signal. Modulation techniques provide high data rate transmission, data security, quality signal, simple architecture, low power consumption, good performance over a fading communication channel, increased channel capability, greater accuracy in the presence of noise and distortion, *etc.* [22]. However, there are a number of tradeoffs in the digital modulation like hard to design complex structured, disadvantages of analog counterparts and bandwidth size. The main criteria for choosing the kind of modulation schemes are based on power, bandwidth and system efficiencies [23,24]. Figure 3 shows the most common modulation used in biomedical devices.

Modulation schemes are chosen or designed according to the channel characteristics in order to optimize their performance. Channels can be classified as additive white Gaussian noise channels (AWGN), band limited channels and fading channels. The AWGN channel is a universal channel model for analyzing modulation schemes by adding the white Gaussian noise to the signal passing through the channel. The band limited channel is limited when it is smaller than the signal bandwidth. The fading channel happens when the amplitude and phase change rapidly over a short period of time.

## 4. ASK Modulation and Demodulation

**Figure 4.**
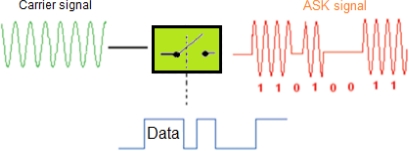
Principle of ASK modulation.

The amplitude shift keying (ASK) or on/off keying (OOK) is the simplest digital modulation used in wireless telemetry bio-devices and biomedical implanted devices [25]. In these types of modulation, no carrier is used during the transmission which minimizes the power consumption of the modulator [26]. The principle of ASK transmission is explained in [27] as shown in Figure 4 and as follows:

(1) $$S_{\mathrm{ASK}}\;(t)=b(t)\times c(t)$$

(2) $$b(t)=\{\begin{array}{c} \sqrt{E_{b}}\;\ldots \;\ldots \;\ldots \;\ldots \;.\;.\;\mathrm{binary}\;1\\ 0\;\ldots \;\ldots \;\ldots \;.\;.\;\mathrm{binary}\;0 \end{array}$$

(3) $$c(t)=\sqrt{\frac{2E_{b}}{T_{b}}}\;\text{cos}\left(\omega_{c}\;t\right)$$

where *b*(*t*) is the binary message, *c*(*t*) is the carrier signal, *E_b_* is the bit energy and *T_b_* is the bit duration.

In general ASK modulation is used in the implanted part for its simplicity and low power consumption. However, it has a number of limitations for high-bandwidth data transmission, high order filters with sharp cut off frequencies and large capacitors cannot be easily integrated for low-frequency RF applications [28]. There are two methods of ASK demodulation: coherent and non-coherent detection, however, most designers have used the non-coherent method due its simplicity and low power consumption [29]. The coherent method uses carrier phase information for detection. This method uses a product detector and a phase-locked beat frequency oscillator for detection. In non-coherent methods no carrier phase is used for detection based on filtering signal energy within allocated spectra and envelope detectors. The performance degradation of the non-coherent method is about 1–3 dB when compared to coherent detection, depending on *E_b_/N_0_*.

Summaries in terms of frequency, data rate, power, size and application of ASK modulator for various telemetric wireless and implanted devices are shown in Table A1.

### 4.1. ASK Modulation for Neural Implants

An electronic ASK demodulator subsystem has been developed by Yu and Najafi using a power-on-reset block, op/am E class, low drop-out regulator and hybrid charge redistribution ADC with 10-bit resolution [30]. However, its implementation suffers from inaccurate synchronization of the clock and data signals. Djemouai designed a ASK demodulator by using a current edge detector circuit and two current mode comparators to stimulate the nerves and muscles or to measure and sense physiological signals [31]. This demodulator can process the input current with a very smal modulation depth and is converted into voltage pulses to control the state of the ouput stage. However, the size of the demodulator is one of the issues. A new CMOS current mode ASK demodulator used to extract and detect digital data of current signals with very small modulation depth of 250 kHz and distinct levels of amplitude variation of 4 μA to 4.5 μA has been reported [22]. To solve the aforementioned problems, Yu and Basirullah [32] proposed an integrated low power clock and data recovery circuit without DLLs for neural recording microsystem. The circuit employs a ASK modulation scheme and pulse position modulation (PPM) with modulation index *m* = 1 to facilitate clock recovery and reduce power dissipation. Recently, a novel low power ASK-PPM receiver was designed using inductive peaking, clock and data recovery circuit with multiple charge pumps to boost up the gain and facilitate the time-to-voltage conversion [33]. In this system, the required voltage reference is generated adaptively to cover a large range of data rates.

### 4.2. ASK Modulation for Cochlear Implants

The electronic system of the modulator and demodulator is replaced by a NAND gate, diode and RC with CMOS transistor to control the carrier signal for cochlear prostheses [34]. This system used five inverters in order to satisfy the constraint of integration and to generate an oscillation operated carrier frequency of 20 MHz. Recently, Yan *et al*. [35] proposed a monostable circuit for low-power CMOS ASK system with clock and data recovery for cochlear implants integrated under 0.18 μm CMOS technology. Pulse width modulation (PWM) signals with delay-locked loops (DLL) are used to recover and resynchronize the clock and data of the system [36]. However, the expense of increased area and overhead power are problems in this system.

### 4.3. ASK Modulation for Complex Implants

Gunnar tested an ASK modulator with 200 Kbps carrier frequency to achieve high small-signal bandwidth with low power levels and this unit worked better for complex implants such as stimulating electrode arrays or visual implants [37]. A high performance and low power consumption unit without capacitor and resistor and with clock recovery circuit of 10 MHz carrier frequency, 0.35 μm CMOS, 2 Mbps data rate and less than 84 μw ASK demodulator was developed for complex implants [29]. The wireless receiver of the ASK demodulator consisted of a current source, a class AB operational amplifiers with common source, a track and latch stage and a Schmitt trigger. To reduce the CMOS size, Li and Zhang [38] developed a novel mixed-signal interface for bio-telemetry complex implantable systemd consisting of a digital processing circuit and analog front-end. The system included power amplifier, ASK demodulator, clock extraction and power recovery without passive elements, *i.e.*, capacitor and resistor. The CMOS size is reduced to 0.18 μm, which is less than that of other complex implant systems and in addition, the power supply is only 1.8 V.

### 4.4. ASK Modulation for Wireless Telemetry and Endoscopic Implants

In wireless capsule endoscopic systems, the data transmission from inside to outside the human body is high to achieve high quality medical images, so for quality internal biomedical imaging, Han *et al.* [39] proposed a pseudo differential stacked class-A power amplifier based on phase lock loop (PLL) and RF conversion circuit. In the system, a 20 MHz, 0.25 μm CMOS, 1 Mbps data rate, 3.62 mm^2^ chip area of 3.17 mA power consumption ASK modulator is used to generate the low frequency carrier by a voltage controlled oscillator and convert the low frequency ASK signal into the 2.4 GHz industrial scientific and medical (ISM) band. However, the sizes of the demodulators are still not up to the level needed. Therefore, after a detailed review of the demodulators like CMOS current mode [40], wireless capsule endoscopic application [39], neuro-stimulus chip for retinal prosthetics, neuromuscular, cochlear devices [36,41–44] and nano-mechanics based C-reactive protein detection [45], Gong *et al.* [46] proposed a self-sampling demodulator without passive elements consisting of a pulse shaper, voltage scalar, level contractor and self-sampler. Again in 2009, Liang *et al.* [47] designed an ASK demodulator for wireless telemetry biomedical applications, which supports data rates up to 300 kbps and detects envelopes with a modulation index of 1% or more. The circuit design includes a single-to-differential OTA, a current mode full-wave rectifier, a log-domain peak detector, a variable-gain amplifier and a comparator.

### 4.5. ASK Modulation for General Implant Applications

To reduce the size of the implanted devices, a low-efficient RF induced capacitor-less, *i.e.*, C-less ASK demodulator was developed to regulate the stable output voltage. The detailed characteristics of the proposed demodulator are shown in [28]. In order to further reduce the size of implantable devices, Lee *et al.* [48,49] developed C-less and R-less without trigger envelope detectors. These demodulators improve the noise margin and also reduce the quantity of transistors. Huang *et al*. [50] also designed a ASK demodulator without passive elements and with only 15 CMOS transistors. The demodulator has a high bandwidth of 300 MHz for lower ISM band frequencies, which is enough for almost all kinds of biomedical implantations. Gong *et al.* [51] developed their previous work [46] further, providing the ability of working over a small modulation index without any passive elements using a self- sampling scheme to reduce the chip area and cost as well as to receive higher efficiency. Kao *et al*. [52] designed an ASK demodulator based on a maximum modulation index up to 2.86% and a 50% maximum modulation rate. The size of the CMOS is comparatively smaller in this general category of biomedical implants. The modulation index and rate are designed as:

(4) $$\mathrm{Modulation Index}=\frac{V_{H}-\left(-V_{L}\right)}{V_{H}+\left(-V_{L}\right)}\times 100\%$$

(5) $$\mathrm{Modulation Rate}=\frac{\mathrm{Data Rate}}{\mathrm{Operated Carrier}}\times 100\%$$

where *V_H_* and *V_L_* represent the maximum and minimum amplitudes of the modulated signal wave form in which the modulation index increases the efficiency of the system and reduces the size. Data rate is the rate at which information is being transferred and operating carrier is the suitable operating frequency in the modulation rate or signaling speeds that contributes to transfer data rate with high efficiency.

To reduce the power consumption in medical implanted devices an ASK demodulator without using any passive elements has been designed [53]. It consists of a rectifier, an envelope detector, digital shaper and load driver. In general, non-coherent ASK modulators are sensitive to the carrier frequency and modulation index. To avoid this disadvantage, a new circuit based comparator is designed in an ASK demodulator including a full wave rectifier, a voltage reference and a small capacitor to filter the high frequency components and a signal formatter circuit [54].

## 5. FSK Modulation and Demodulation

FSK is one of earliest and most suitable modulation techniques for digital modulation. The principle of FSK modulation is to send binary data with two different frequencies and the resultant modulated signal is regarded as amplitude modulation (AM) of different carrier frequencies. There are two types of FSK modulation: *i.e.*, non-coherent or discontinuous and coherent FSK modulation [55]. In the non-coherent types of FSK modulation different frequencies is represented by binary 1 and 0 as follows:

(6) $$\begin{array}{l} S_{1}\;(t)=A\;\text{cos}\;\left(2\pi f_{1}\;t+\phi_{1}\right),\;\mathrm{kT}\leq t\leq \;(k+1)T,\;\mathrm{for}\;1\\ S_{2}\;(t)=A\;\text{cos}\left(2\pi f_{2}\;t+\phi_{2}\right),\;\mathrm{kT}\leq t\leq \;(k+1)T,\;\mathrm{for}\;0 \end{array}$$

where *ϕ*_1_ and *ϕ*_2_ are initial phases at *t* = 0 and they are not the same in general. *T* is the bit period of the binary data. This type can be generated by switching the modulator output line between two different oscillators. However, in the coherent types of FSK modulation, the two coherent signals have the same initial phase *ϕ* at *t* = 0 as follows:

(7) $$\begin{array}{l} S_{1}\;(t)=A\;\text{cos}\left(2\pi f_{1}\;t+\phi \right),\;\mathrm{kT}\leq t\leq \;(k+1)T,\;\mathrm{for}\;1\\ S_{2}\;(t)=A\;\text{cos}\left(2\pi f_{2}\;t+\phi \right),\;\mathrm{kT}\leq t\leq \;(k+1)T,\;\mathrm{for}\;0 \end{array}$$

In this section, a detailed summary of FSK modulation in terms of frequency, data rate, power, sizes and applications of various telemetric wireless and implanted devices is listed in Table A2.

### 5.1. FSK Modulation for Biological Implants

It is usually necessary for a nurse to be near a patient all the time for monitoring and watching the patient’s condition. To avoid this situation, a new kind of low cost monitoring system is developed [56] that consists of a transmitter and receiver. The transmitter is located near the patient or can be attached to him and the receiver located in the monitoring room to be watched and monitored by the physician and the nurses. The multi-signal of the system is modulated by analog FM modulation and FSK carrier frequencies at 447 MHz. However, the system suffers from interfacing between the signals and noise. To address the above issues, a low-power FSK modulator and demodulator for biological signals can be used to reduce the noise. The transmitter side of the system can also be used a four stage differential ring VCO with tone frequency located at 402–405 MHz carrier frequency.

### 5.2. FSK Modulation for General Implants

Much research has been focused on various types of biomedical implant devices. In this regards, Ghovanloo and Najafi [57] developed an FSK protocol and compared it with the ASK protocol. The developed high rate data transfer protocol receives the serial bit from the input clock, extracted from 2 MHz to 20 MHz FSK carrier signal. The extracted FSK signal is as follows:

(8) $$f(t)=f_{0}\;(t)\;\text{sin}\left(2\pi f_{0}\;t+\phi \right)+f_{1}\;(t)\;\text{sin}\left(2\pi f_{1}\;t+\phi \right)$$

A novel 4-FSK demodulator technique was used to improve the bit error rate (BER) performance and to increase the decision accuracy of the system by generating additional zero-crossing using a modified ZIFZCO with clocks [58]. However, this device has relative high power consumption. Ghovanloo and Najafi [59] further developed this work in [57] using the same carrier frequency to reduce the power consumption and chip area. The demodulator circuit of the system is developed with a data rate to carrier frequency ratio up to 67% to require a much lesser power consumption of 0.38 mW. Again, Ghovanloo and Najafi [60] developed a three FSK demodulator circuit with data-rate up to carrier-frequency ratio up to 67% in 5–10 MHz. This novel method was used to send data inductively to a wireless biomedical implant with 4 Mbps data rates. The power consumption and the chip area of the implanted devices are comparatively less than that of the previous system.

CMOS technology is very popular for designing an effective modulator. Accordingly, Sodagar and and Najafi [61] reviewed many of the implanted circuits and devices that used standard CMOS technologies. Tekin *et al*. [62] have developed a low power FSK modulator/demodulator for an MICS band transceiver to reduce the CMOS size and chip area. Wend *et al*. [63] developed two different types of demodulators such as an analogue FSK (AFSK) and a digital FSK (DFSK) for general implanted devices. The AFSK consists of a signal balance mixer and a comparator, however, the DFSK consists of only one block. Both demodulators are implemented in 0.18 μm CMOS technology with a power supply of 1 V. The AFSK demodulator occupies a chip area of 0.016 mm^2^ and the data rate is 2.5 Mbps. The DFSK occupies a chip area of 0.004 mm^2^ and the data rate is up to 5 Mbps. The power consumption of ASFK and DFSK demodulators are 0.47 mW and 0.022 mW, respectively.

### 5.3. FSK Modulation for Physiological Implants

To monitor the various physiological parameters inside the human body, the transmitter unit sends the sensor data outside the human body as explained by Zhu *et al.* [64]. This paper proposed a new low-power (FSK) modulator for biomedical sensors. The new circuit integrates the modulation functionality into the oscillator itself by using the data signal to control the oscillation frequency, and the circuit can generate a tunable carrier frequency for different data signals. The author compared his work with another works such as Mohseni *et al*. [65], which provided one silicon chip which combines single-channel and multi channel with lower dissipation. Harrison *et al*. [66] developed a prototype integrated circuit for wireless neural recording from 100-channel microelectrode. Haider *et al*. [67] proposed a system consisting of a FSK generator block and data generator used for low-voltage and low-power operation. However, Zhu *et al.* [64] developed a FSK modulator for biomedical sensor circuits that has better properties for physiological implants.

## 6. PSK Modulation and Demodulation

Phase shift keying (PSK) constitutes a large class of digital modulation techniques. In the last decades PSK modulation has been widely used in wireless communication for biomedical modulation techniques. This review includes the important research which has used PSK modulation in biomedical applications. In PSK modulation, correlated or matched filter implementation of the coherent detector are compared with a reference signal. The reference signal is generated by the carrier recovery circuit, which is synchronous to the received signal in terms of frequency and phase. However, the differential coherent demodulator is non-coherent in the sense that phase coherent reference signals are not required, which is used to overcome the adversary effect of the random phase in the received signal. The most common digital schemes appropriate for biomedical data transmission are briefly described as PSK, binary PSK (BPSK), differential PSK (DPSK), quadrature PSK (QPSK), differential quadrature PSK (DQPSK), offset quadrature PSK (OQPSK) and quasi-coherent PSK (QCPSK) in [68,69], as shown in Table A3.

### 6.1. PSK Modulation for Various Stimulator Implants

A stimulator is an agent that excites some functional activity of the human body. Accordingly, Hu and Sawan [70] developed a fully integrated neuromuscular stimulator with a BPSK demodulator using COSTAS loop topology. The demodulator consists of comparator, multipliers, loop filters, VCO and 90° phase shifter. The BPSK demodulator can be modified as a QPSK demodulator to get double the data transfer rate. These types of demodulators are complex in function. To reduce the complexity of the system and to achieve low power and high data rate transmission, Lu and Sawan [71] designed a system with two modulators and two demodulators using OQPSK modulation. The circuit was integrated in 0.18 μm CMOS technology and operated with 13.56 MHz carrier frequency to achieve 8 Mbps data rates, *i.e.*, very fast transmission. The power consumption of the modulators and demodulators is relative low. The BPSK modulation was also used to transmit data and power from the external part to the implant and the load shift keying (LSK) modulation is used to transmit the received data from the implant to the external part [72]. The implanted device consists of a BPSK demodulator that functions as a brain stimulator-based PLL. Lee *et al*. [73] proposed a bio-device for transcutaneous wireless telemetry using the near-field inductive coupling technique with round-wire coils for a cardiac micro stimulator. PSK modulation is used to decode the transmission data.

### 6.2. PSK Modulation for General Implants

To reduce the size and power consumption, Hu and Sawan [74] further developed their previous work keeping the same parameters in [70] but using BPSK modulation for general body implants. The BPSK demodulators in [70,71] were further developed by Deng *et al*. [75] using QPSK modulation and modified COSTAS loop technology. The program was simulated using same carrier frequency of 13.56 MHz with a data rate of 4 Mbps, *i.e.*, faster data transmission. Again, a BPSK demodulator is designed using a phase frequency detector based PLL for robust performance [76]. Two different circuit implementationa for demodulator with a carrier frequency of 13.56 MHz are used in this system.

### 6.3. PSK Modulation for Neural Implants

Most implantable devices use an inductive coupling link to transmit both power and data between coils using the same carrier frequency. The system circuit configuration is complex. Zhou *et al*. [77] separated the power and data, then transmitted them with different frequencies using DPSK modulation employing two transmitters and two receivers. The system power is transmitted with 1 MHz carrier frequency and the data is transmitted with 20 MHz carrier frequency. This method made the system simpler by reducing the circuit complexity. However, the as-developed system is big in size and has higher power consumption compared with other systems. The model of the system is as follows:

(9) $$\omega_{1}=\frac{1}{\sqrt{L_{1}\;C_{1}}}\;\mathrm{and}\;\omega_{2}=\frac{1}{\sqrt{L_{2}\;C_{2}}}\;\mathrm{when}\;k=0$$

(10) $$\omega_{1}=\frac{1}{\sqrt{L_{1}\;C_{1}}+L_{2}\;C_{2}}\;\mathrm{and}\;\omega_{2}=\infty \;\mathrm{when}\;k=1$$

where *ω*_1_ and *ω*_2_ are frequency components of the two resonant tanks, respectively. *L_1_* and *L*_2_ are the inductances and *C*_1_ and *C*_2_ are the capacitances of a linearized model coupling between two resonant tanks, respectively. K is the coupling coefficient between coils that results in frequencies splitting away from their original values. When *k* ≠ *0*, both resonant tanks will contain frequency components *ω*_1_ and ω_2_. However, no interference is present at *k* = 0, *i.e.*, the power and data resonant tanks remain at their original frequencies. When *k* = 1, the upper frequency shifts to infinity. Again, when *L_1_* >> *L_2_* and *ω*_1_ ≈ ω_2_, the frequency of the power resonant tank is hardly affected due to the small amount of interference from the data transmitter.

A non-coherent PSK receiver without PLL was developed by using a band pass sampling theory for neural implants [78]. Zhou *et al.* [79] further developed his prior system [77] using a DPSK modulation in the analog domain to cancel the interference signal for a dual-band. This system is able to cancel 12 dB interferences signals without using complex filters. The system still suffered from high power consumption. In general, wireless powered signal-to-noise ratio (SNR) of the received signal is high for the short-range in neural implants. To overcome this situation, Gong *et al*. [80] proposed a quasi-coherent PSK (QCPSK) demodulator employing a direct carrier processing to extract the timing signals.

In order to eliminate the power interference in the data receiver in neural implants, a fully integrated DPSK is used for high density biomedical implants through dual-band telemetry [81]. The carrier frequency of the power and data rate are separated within a minimum sampling rate which results in reduced chip area and makes it possible for all blocks to be implemented in one chip. This system suffers from relatively high power consumption. Power consumption is an essential key in biomedical implantable devices. To achieve reduced power consumption, a BPSK demodulator based on a digital-analog hybrid COSTAS loop and a digital multiplier is proposed in [82]. The digital multiplier is used in place of an analog multiplier to reduce the power consumption.

### 6.4. PSK Modulation for Complex Implants

Complex implants are very sensitive to data transmission problems. In this regard, a new fully digital BPSK demodulator and load shift keying (LSK) has been used in complex implanted devices to reduce the power consumption and increase the data transmission rate [83]. The back telemetry technique is used in this work and verified through simulation and measurement. The obtained results show that the system improves the data transmission rate compared to conventional bi-level back telemetry. Furthermore, Xu *et al*. [84] improved their previous work in [83] by using a fully digital BPSK demodulator based on digital PLL and a multi level LSK modulator to generate the incoming BPSK signal data for complex implants with higher data rate back telemetry.

Retinal and cochlear complex implants require high speed data transfer. A novel BPSK data demodulator and clock recovery circuit based on noncoherence is developed in [85,86] in which the developed circuit works with the outstanding data-to-carrier-frequency ratio of 100%. The demodulator powered the implanted device with high data rate and low power consumption, as can be seen in Table A3. The demodulator is implemented in a 0.18 μm CMOS process with 10–20 MHz carrier frequency. The power consumption is only 119–310 μW with this 10–20 Mbps data rate, which are a lower power consumption and higher data rate compared to the previous work.

The analysis, investigation and experimental measurements for high data rates have been developed using FPGA/CLPC programming [87]. The n-PSK demodulator was programmed with VHDL to generate digital binary quadrature and eight PSK signals. In this system the carrier frequency was chosen to be 135 kHz operated by inductively coupled RF power and a modified hybrid class E and F power amplifier was used to simulate the system. Asgarian and Sodagar [88] proposed his further designed modulator based on QPSK using FPGA/CPLD for implantable telemetry applications. The local clock oscillator is used at 25.175 MHZ with a carrier frequency of 12.5 MHZ. This proposed design has low power consumption and is small in size, which makes it suitable for biomedical applications. To improve the high data rate transmission and reduced the system complexity, Asgarian and Sodagar [89] proposed a phase-silence-shift keying (PSSK) modulation. PSSK modulation needs double the bandwidth of PSK modulation.

## 7. Current Challenges and Problems

Biomedical device technology has been recently used for medical applications in the human body by monitoring or recording many signals such as EMG, ECG, EOG and ERG and monitoring or diagnosing the current conditions of implantable devices such as retinal implants, cochlear implants, pacemakers, brain implants and for wireless capsule endoscopy. Two of the most important issues for biomedical implants are patients’ safety and comfort. These can be achieved by reducing the power consumption, switching losses, choosing suitable modulation and carrier frequency according to a MICS table and by increasing the efficient energy transfer capabilities, for example, using near-field wireless energy transfer that results in electromagnetic fields that would not heat the tissues. However, for example, heating of pacemaker leads is the major problem undermining magnetic resonance imaging. Therefore, hundreds of patients with a pacemaker or implantable cardioverter face safety and reliability issues [90,91]. Typically a wireless biomedical devices consists of two parts, the external part that transmits RF signals to the internal part through an inductive coupling downlink using variety of modulated signals such as ASK, FSK, PSK (BPSK, QPSK, DBPSK, DQPSK, OQPSK), and it must transmit with high efficiency and low power consumption. Thus, an aim of research into implantable biomedical devices is to develop such devices with low power consumption, efficient energy transfer, the highest data rate, efficient power amplifiers, small size and low-cost, so many parameters need to be considered, as discussed in the following sections.

### 7.1. Adopting the Proper Method to Choose the Suitable Modulation Used for the Wireless Telemetry

Adopting aproper method to choose the suitable modulation to be used for the wireless telemetry depends on the nature and type of the implanted device. Some implanted devices such as brain, retinal, cochlear implants, and wireless capsule endoscopy require high data rate transmission [35,38,39]. Thus, the advantages and disadvantages of the suitable modulations used in biomedical devices and biotelemetry systems need to be focused. For example, the BPSK modulation offers a practical circuit implementation by transmitting 1-bit binary data by the influence of shifted carrier frequency, which does not change the phase carrier of the sine wave. However, there is a change in the carrier phase to a negative sine wave for 0-bit. Thus, the BPSK is only able to modulate 1-bit/symbol, which may not provide a high enough data rate due to the bandwidth limitations and and the demodulator power consumption [27]. These problems can be solved by techniques offering high data rates like QPSK and n-PSK modulation. However, these suffer from complicated structures and high power consumption, as explained in Section 6. The FSK is general uses two signals with different frequencies to represent binary 1 and 0. These modulations offer high date rates and suffered from complications and size issues [22,26] as explained in Section 6. The PSK and FSK are used for implanted devices which need high data rates such as retinal and cochlear implants. We have seen that ASK modulation is more suitable than FSK and PSK for implant devices and telemetry systems within a short range. Thus, modulation with simple hardware implementation and small in size, fast and reliable transmission, less power consumption and constant RF signal would be the best choice for a wireless telemetry system.

### 7.2. Suitable Carrier Frequency

The carrier frequency is very important in designing implanted devices and biotelemetry systems. Most of the implantable devices are powered by low frequency, less than 1 MHz, however, the standard safety levels with respect to human body exposure to radio frequency electromagnetic fields is 3 kHz–30 GHz [4]. The standards frequencies according to the MICS are specified between the frequencies of 402–405 MHz, which involves a number of allowable frequencies such as 27 MHz. The second standard is the ISM standard dealing with 13.56 MHz band. The RFID standard deals with 125–135 kHz for low frequency bands. The low and high frequencies between 3 kHz–30 MHz are widely used in transcutaneous wireless due to its ability penetrate water and skin over a short range.

### 7.3. Suitable Power and Class Amplifier

The power amplifier plays an important role in low and high frequency bands to amplify the signal to a certain coupling coil which transmits the power and data to the implantable devices. Thus, an implant system needs a highly efficient power amplifier. The suitable class of amplifier is very important to transmit a stable RF signal with high efficiency compared to these conventional amplifiers such as class A, B, C [92]. In general, class E and F amplifiers have the same properties and are widely used in implantable devices and telemetry systems. The E class amplifier requires only one active device within kHz to several MHz, in which shifting resonance frequency decreases the output power transmission. Thus, an automatic frequency correction circuit is needed for high rates of data transmission. However, F class amplifiers require at least two active devices, which increases the power consumption and size. The frequency is limited in low frequencies due to its switching losses. Thus, higher frequencies and inductively coupled system are suitable for better response and comparatively efficient even in the case of frequency shifting.

### 7.4. Coils Used in the Wireless Telemetry System

The wireless inductive coupling use the magnetic field to transfer data and power from the external part to the internal part. In general, RF short-range communication transmits a low power irradiated from the reader coil antenna to offer fixed sinusoidal carrier amplitude that provides a stable wireless transfer power. The bio-device system is composed by a primary coil integrated and isolated inside the human body and a secondary coil located outside the body. In most cases, the primary coil is tuned in series resonance to provide a low impedance load for driving the transmitter coil, whereas the secondary coil is almost invariably in parallel. Thus, to have better power transfer efficiency of inductive coupling link both sides of the link are tuned at the same resonant frequency and the stability of the RF signal need to provide a high readability at the implant device in terms of the distances from the reader coil.

### 7.5. Size of the Implanted Devices

Implantable devices need to be as small as possible, so as to be less invasive for the human body. The batteries in biomedical implants have several problems such as limited life time, chemical side effects and being large in size. Researchers are trying to eliminate the use of batteries from implanted devices in the human body and have proposed various solutions to power implantable devices such as using inductive coupling link, power harvesting with piezoelectric material and body motion. The silicon area should be as small as possible to minimize external components that can reduce the overall size of implantable devices. Reducing the complexity of the electronic block circuits and the number of the passive elements such as capacitors and resistors would help to reduce the size of the implanted devices. This can be achieved by developing techniques and methods such as auto-zero technique [93], FPGA methods [86] and artificial intelligence methods [94].

### 7.6. Low-Cost and Simplest Design

In the 1950s, when the first biomedical implantable devices was implanted, the focus was on scientific success, and the economic aspectd were not important at that time. However, with the increasing use of the biomedical implantable devices, the economy of the implantable devices is very important. in addition to safety and comfort for the patient. Thus, low-cost and simple designs became an important factor and challenges for the designers.

## 8. Conclusions and Suggestions

This article outlines the various modulation techniques and their suitability for wireless data transmission for bio-medical implanted devices. The different modulation schemes show different types of bandwidth data transmission and frequencies. The ASK modulation is based on on/off keying, multiplication of the binary message and the carrier signal used for low power consumption, while the FSK mode is the most suitable digital modulation, which sends binary data with two different frequencies and the resultant modulated signal is represented by binary 1 and 0. PSK is the largest class of digital modulation applied in complex bio-medical implanted devices by coupling inductive link to transmit both power and data between coils using the same carrier frequency. The review investigated and observed that the quality of the implanted devices and their reliability depends on the modulation scheme in terms of data transmission, operating frequency and power consumption. The main challenges in existing biomedical devices are the data transfer rated, suitable modulation and carrier frequency for wireless transmission, size, cost and design. Highly efficient systems with minimum error, and enhanced encoding of information to protect the data acquired from the human body must be designed. Thus, choosing a suitable modulating technique is very important to achieve high data transfer rates according to the implanted applications. This study explained the existing implantable biomedical devices, their characteristics, challenges and problems in aiming to develop low power consumption, efficient energy transfer, highest data rate, efficient power amplifiers, small size and low-cost, reliable future biomedical implantable devices and their modulation techniques. To achieve the aforesaid aims, advanced techniques like artificial intelligence can be use to provide a stable RF signal and offers flexibility for updating the data to the implanted devices using automatic frequency control programs. This research will lead the increasing need to develop new and improved implanted and wireless telemetry devices.

## Appendix

**Table A1.** ASK modulations used in telemetric wireless and implanted devices.

**Technique**	**Carrier (MH_Z_)**	**CMOS (μm)**	**Data Rate (Mbps)**	**Power consumption**	**Power Supply (V)**	**Chip area (mm^2^)**	**Applications**	**Year [ref]**
ASK	-	0.8	-	0.47–1.963 mW	-	4.82	Neural system	2003 [30]
ASK	250	0.18	-	-	1.8	-	Phyiological signal	2004 [31]
ASK	1	0.6	0.004–0.018	70 μW	2.7	-	Neural System	2006 [32]
ASK	1.5	0.25	0.007–0.045	31 μW	2.5	-	Neural System	2010 [33]
ASK	20	0.35	0.001	0.062 μW	-	-	Cochlear implant	2004 [34]
ASK	10	0.18	0.500	29.52 μW	-	-	Cochlear Implants	2010 [35]
ASK	10	0.5	0.200	60 μW	3.3	-	Complex implants	2000 [37]
ASK	10	0.35	2	<84 μW	3.3	-	Complex implants	2006 [29]
ASK	10	0.18	1	2.75 mW	1.8	-	Complex implants	2008 [38]
ASK	20	0.25	1	3.17 mA	2.5	3.62	Endoscope system	2006 [39]
ASK	0.250	0.18	-	-	1.8	-	Nerves system	2004 [40]
ASK	2	0.18	1	0.5 mW	1.2	-	Wireless sensor	2006 [46]
ASK	4	0.18	0.300	1 mW	1.8	5.04	Telemetry Applications	2009 [47]
ASK	2	0.35	0.010	10.234 mW	3.2-3.4	-	General	2004 [28]
ASK	2	0.35	-	1.01 mW	3.3	0.0030	General	2007 [48]
ASK	2	o.35	0.250	1.01 mW	3.3	0.0030	General	2008 [49]
ASK	300	0.35	1	0.58 μW	2.5–2.8	3.2	General	2008 [50]
ASK	2	0.18	1	0.336 mW	-	-	General	2008 [51]
ASK	2	0.18	1	o.396 mW	1.8	3.6e-3	General	2009 [52]
ASK	13.56	0.35	1.2	0.306 mW	-	3.2	General	2010 [53]
ASK	10	0.35	1	-	3–4.2	-	General	2011 [54]

**Table A2.** FSK modulations used in telemetric wireless and implanted devices.

**Technique**	**Operated Carrier (MH_Z_)**	**CMOS Technology (μm)**	**Data Rate (Mbps)**	**Power consumption**	**Power Supply**	**Chip area (mm^2^)**	**Applications**	**Year [Ref]**
FSK	447	-	-	-	Low battery	-	ECG	2000 [56]
FSK	2–20	1.5	1	0.55 mW	5	0.67	General	2002 [57]
FSK	-	0.35	-	3 mW	3	0.78	General	2002 [58]
FSK	2–20	1.5	2	0.5 mW	5	0.41	General	2003 [59]
FSK	5–10	1.5	4	0.38 mW	5	0.29	General	2004 [60]
FSK	402–405	0.18	-	33.41 μA	1.5	-	Biological signal	2006 [62]
AFSK	22/5	0.18	2.5	0.47 mW	1	0.016	General	2007 [63]
DFSK	5/10	0.18	5	0.022 mW	1	0.004	General	2007 [63]
FSK	6.459	0.35	450 kbps–Mbps	0.02 mW	2.5	0.003	Physiological sensors	2010 [64]
FSK	88–108	1.5	0.001	-	1.5	0.21	Physiological sensors	2005 [65]
FSK	433	0.5	0.330	0.550–1.2 mW	3.3	0.39	Physiological sensors	2007 [66]
FSK	0.536	0.5	0.250	0.431 mW	3	-	Physiological sensors	2008 [67]

**Table A3.** PSK modulations used in telemetric wireless and implanted devices.

**Technique**	**Operated Carrier (MHZ)**	**CMOS Technology (μm)**	**Data Rate (Mbps)**	**Power consumption**	**Power Supply**	**Chip area (mm^2^)**	**Applications**	**Year [ref]**
BPSK	13.56	0.18	1.12	0.414 mW	1.8	400 × 370	Neuromuscular stimulator	2004 [70]
OQPSK	13.56	0.18	8	-	1.8	-	Neuromuscular stimulator	2008 [71 ]
BPSK	13.56	0.5	0.1–1.69	5 mW	3.3	0.1	Brain stimulator	2008 [72]
PSK	0.250	0.35	60 bpm	31.5 μW	5	1.5 × 1.6	Cardiac stimulator	2011 [73]
BPSK	13.56	0.18	1.51	0.610 mW	1.8	0.19	General	2005 [74]
QPSK	13.56	0.18	4	0.75 mW	1.8	-	General	2006 [75]
BPSK	13.56	0.5	0.020	3 mW	-	1	General	2008 [76]
DPSK	20	-	1–2	6 mW	-	-	Neural System	2006 [77]
PSK	Multi frequency	0.35	1–2	6.2 mW	-	2.6 × 1.7	Neural System	2007 [78]
DPSK	20	0.35	1–2	6.2 mW	-	2.6 × 1.7	Neural System	2008 [79]
QCPSK	-	0.18	0.800	0.059 mW	1.8–3.3	0.005	Neural prosthesis	2008 [80]
DPSK	22	0.35	4	8.4 mW	-	1.4 × 1	Neural Implants	2008 [81]
BPSK	10	0.35	2	485.4 μW	3.3	0.17	Neural System	2010 [82]
BPSK–LSK	13.56	0.5	0.800	2.3 mW	-	-	Complex System	2008 [83]
BPSK–LSK	13.56	0.5	-	2.3 mW	1.65–3.3	536 × 546 μm^2^	Complex System	2009 [84]
BPSK	10	0.18	20	310 μW	1.8	-	Complex System	2009 [85]
BPSK	10	0.18	10	119 μW	1.8	-	visual prostheses	2010 [86]

## References

[1] Rhees D.J. (2009). From Frankenstein to the Pacemaker: A Profile of the Bakken Museum. IEEE Eng. Med. Biol. Mag.

[2] Yekeh K., Kohno R. Wireless Communications for Body Implanted Medical Devices.

[3] Madal S., Sarpeshkar R. (2008). Power-Efficient Impedance-Modulation Wireless Data Links for Biomedical Implants. IEEE Trans. Biomed. Circ. Syst.

[4] Lazzi G. (2005). Thermal Effects of Bio-Implants. IEEE Eng. Med. Biol. Mag.

[5] Kathuroju P.K., Jampana N. (2009). Effect of Low Frequency Pulsed DC on Human Skin *In Vivo*: Resistance Studies in Reverse Lontophoresis. Sens. Transduc. J.

[6] Asgarian F., Sodagar A.M. A Low-Power Non Coherent BPSK Demodulator and Clock Recovery Circuit for High-Data-Rate Biomedical Applications.

[7] Miranda H., Gilja V., Chestek C.A., Shenoy K.V., Meng T.H. (2010). HermesD: A high-Rate Long-Range Wireless Transmission System for Simultaneous Multichannel Neural Recording Applications. IEEE Trans. Biomed. Circ. Syst.

[8] Atluri S., Ghovanloo M. Incorporating Back Telemetry in a Full-Wave CMOS Rectifier for RFID and Biomedical Applications.

[9] Soria S., Berneschi S., Brenci M., Cosi F., Nunzi Conti G., Pelli S., Righini G.C. (2011). Optical Microspherical Resonators for Biomedical Sensing. Sensors.

[10] Bhoir D.V., Panse M. (2009). Advances in Cochlear Implant Implementation. Int. J. Recent Trend. Eng.

[11] Otal B., Alonso L., Verikoukis C. (2011). Energy Efficiency Analysis of a Distributed Queuing Medium Access Control Protocol for Biomedical Wireless Sensor Networks in Saturation Conditions. Sensors.

[12] Verma N., Lee K.H., Shoeb A. (2011). Data-Driven Approaches for Computation in Intelligent Biomedical Devices: A Case Study of EEG Monitoring for Chronic Seizure Detection. J. Low Power Electron. Appl.

[13] Chen C.-A., Chen S.-L., Huang H.-Y., Luo C.-H. (2011). An Asynchronous Multi-Sensor Micro Control Unit for Wireless Body Sensor Networks (WBSNs). Sensors.

[14] Kopparthi S., Ajmera P.K. Power Delivery for Remotely Located Microsystems.

[15] Ma Q., Haider M.R., Song Y., Islam S.K. Power-Oscillator Based High Efficiency Inductive Power-Link for Transcutaneous Power Transmission.

[16] Si P., Hu A.P., Malpas S., Budgett D. (2008). A Frequency Control Method for Regulating Wireless Power to Implantable Devices. IEEE Trans. Biomed. Circ. Syst.

[17] Chen H., Liu M., Chen J., Wang Z. (2009). Power Harvesting Using PZT Ceramics Embedded in Orthopedic Implants. IEEE Trans. Ultras. Ferroelectr. Freq. Control.

[18] Zeng H., Zhao Y. (2011). Sensing Movement: Microsensors for Body Motion Measurement. Sensors.

[19] Baporikar V., Karmore S. (2009). Wireless Sensor Network for Brain Computer Interface. Int. J. Adv. Eng. Sci. Tech.

[20] Inoue K., Shiba K., Shu E., Koshiji K., Tsukahara K., Ohu-mi T., Masuzawa T., Tatsumi E., Taenaka Y., Takano H. Transcutaneous Optical Telemetry System Investigation on Deviation Characteristics.

[21] DeHennis A.D., Wise K.D. (2005). A Wireless Microsystem for the Remote Sensing of Pressure, Temperature, and Relative Humidity. J. Microelectromech. Syst.

[22] Ziemer R.E., Tranter W.H. (2010). Principles of Communications Systems, Modulation, and Noise.

[23] Klaus F. (2003). RFID Handbook: Fundamentals and Applications in Contactless Smart Cards and Identification.

[24] Zierhofer C.M., Hochmair-Desoyer I.J., Hochmair E.S. (1995). Electronic Design of a Cochlear Implantant for Multi-Channel High Rate Pulsatle Stimulation Strategies. IEEE Trans. Rehab. Eng.

[25] Cho J.-H., Min K.-W., Kim S. (2006). An ASK Modulator and Antenna Driver for 13.56 MHz RFID Readers and NFC Devices. IEICE Trans. Commun.

[26] Xiong F. (2003). M-Ary Amplitude Shift Keying OFDM System. IEEE Trans. Commun.

[27] Taub H., Schilling D.L. (1999). Principle of Communication System.

[28] Wang C.-C., Hsueh Y.-H., Chio U.F., Hsiao Y.-T. A C-Less ASK Demodulator Implantable Neural Interfacing.

[29] Li H., Li W. A High-Performance ASK Demodulator for Wireless Recovery System.

[30] Yu H, Najafi K. Low-Power Interface Circuits for Bio-Implantable Microsystems.

[31] Djemouai A., Sawan M. New CMOS Current-Mode Amplitude Shift Keying Demodulator (ASKD) Dedicated for Implantable Electronic Devices.

[32] Yu H., Basirullah R. A Low Power ASK Clock and Data Recovery Circuit for Wireless Implantable Electronics.

[33] Yu H., Li Y., Jiang L., Ji Z. A 31 μw ASK Clock and Data Recovery Circuit for Wireless Implantable System.

[34] Naghmouchi F., Ghorbel M., Hamida A.B., Samet M. CMOS ASK System Modulation Dedicated to Cochler Prosthesis.

[35] Yan H., Wu D.-C., Liu Y., Wang D.-H., Hou C.-H. A Low-Power CMOS ASK Clock and Data Recovery Circuit for Cochlear Implants.

[36] Liu W., Vichienchom K., Clements M., DeMarco S.C., Hughes C., McGucken E., Humayun M.S., De Juan E., Weiland J.D., Greenberg R. (2000). A Neuro-Stimulus Chip with Telemetry Unit for Retinal Prosthetic Device. IEEE J. Solid State Circuits.

[37] Gudnason G. A Low-Power ASK Demodulator for Inductively Coupled Implantable Electronics.

[38] Li H., Zhang Y. Low Power Wireless Receiver in CMOS Mixed-Signal for Bio-Telemetry Implantable System.

[39] Han S., Chi B., Wang Z. 8.0-mW 1-Mbps ASK Transmitter for Wireless Capsule Endoscope Applications.

[40] Djemouai A., Sawan M. Integrated ASK Demodulator Dedicated to Implantable Electronic Devices.

[41] Gudnason G., Bruun E., Haugland M. (1999). A Chip for an Implantable Neural Stimulator. Analog Integr. Circ. Sign. Process.

[42] Yu H., Najafi K. Circuitry for a Wireless Microsystem for Neural Recording Microprobes.

[43] Dong M., Zhang C., Wang Z., Li D. A Neurostimulus Chip with Telemetry Unit for Cochlear Implant.

[44] Lee S.-Y., Lee S.-C. (2005). An Implantable Wireless Bidirectional Communication Microstimulator for Neuromuscular Stimulation. IEEE Trans. Circ. Syst.

[45] Chen C.-H., Hwang R.-Z., Huang L.-S., Lin S., Chen H.-C., Yang Y.-C., Lin Y.-T., Yu S.-A., Wang Y.-H., Chou N.-K., Lu S.-S. (2009). A Wireless Bio-MEMS Sensor for C-Reactive Protein Detection Based on Nanomechanics. IEEE Trans. Biomed. Eng.

[46] Gong C.-S.A., Wu C.-L., Ho S.-Y., Chen T.-Y., Huang J.-C., Su C.-W., Su C.-H., Chang Y., Cheng K.-H., Lo Y.-L., Shiue M.-T. Design of Self-Sampling Based ASK Demodulator for Implantable Microsystem.

[47] Liang B., Yang Z., Liu W. An ASK Demodulator for Data Telemetry in Biomedical Application.

[48] Lee T.-J., Lee C.-L., Ciou Y.-J., Huang C.-C., Wang C.-C. C-Less and R-Less Low-Frequency ASK Demodulator for Wireless Implantable Devices.

[49] Lee T.-J., Lee C.-L., Ciou Y.-J., Huang C.-C., Wang C.-C. (2008). All-MOS ASK Demodulator for Low-Frequency Applications. IEEE Trans. Circ. Syst.

[50] Huang C.-C., Chen C.-C, Wang C.-C. R-Less and C-Less Self-Sampled ASK Demodulator for Lower ISM Band Applications.

[51] Gong C.-S.A., Yao K.-W., Chen T.-Y., Chang Y., Su C.-H. (2008). Truly Low-Cost High-Efficiency ASK Demodulator Based on Self-Sampling Scheme for Bio-Implantable Applications. IEEE Trans. Circ. Syst.

[52] Kao C.-H., Tang K.-T. Wireless Power and Data Transmission with ASK Demodulator and Power Regulator for a Biomedical Implantable SOC.

[53] Wang C.-C., Chen C.-L., Kuo R.-C., Shmilovitz D. (2010). Self-Sampled All-MOS ASK Demodulator for Lower ISM Band Applications. IEEE Trans. Circ. Syst.

[54] Daoud D., Ghorbel M., Ben H.A., Tomas J. Fully Integrated CMOS Data and Clock Recovery for Wireless Biomedical Implants.

[55] Xiong F., Proakis J. (2002). Digital Phase Modulation and Demodulation. Encyclopedia of Telecommunications.

[56] Lee H.-K., Park D.-C. Bio-Medical FM-FM-FSK Radio Telemetry System for Multi-Signal Transmission.

[57] Ghovanloo M., Najafi K. A High-Rate Frequency Shift Keying Demodulator Chip for Wireless Biomedical Implants.

[58] Chen Z., Zhang Z., Lau J. A CMOS 2- and 4-FSK Demodulator for Direct-Conversion Radio Paging Receivers.

[59] Ghovanloo M., Najafi K. (2004). Fully Integrated Wideband High-Current Rectifiers for Inductively Powered Devices. IEEE J. Solid State Circ.

[60] Ghovanloo M., Najafi K. (2004). A Wideband Frequency-Shift Keying Wireless Link for Inductively Powered Biomedical Implants. IEEE Trans. Circ. Syst.

[61] Sodagar A.M., Najafi K. Wireless Interfaces for Implantable Biomedical Microsystems.

[62] Tekin A., Yuce M.R., Shabani J., Liu W. A Low-Power FSK Modulator/Demodulator for an MICS Band Transceiver.

[63] Wend R.-M., Li S.-Y., Wang J.-C. Low Power Frequency-Shift Keying Demodulators for Biomedical Implants.

[64] Zhu K., Haider M.R., Yuan S., Islam S.K. A Sub-1 μA Low-Power FSK Modulator for Biomedical Sensor Circuits.

[65] Mohseni P., Najafi K., Eliades S.J., Wang X. (2005). Wireless Multichannel Biopotential Recording Using an Integrated FM Telemetry Circuit. IEEE Trans. Neural Syst. Rehab. Eng.

[66] Harrison R.R., Watkins P.T., Kier R.J., Lovejoy R.O., Black D.J., Greger B., Solzbacher F. (2007). A Low-Power Integrated Circuit for a Wireless 100-Electrode Neural Recording System. IEEE J. Solid State Circ.

[67] Haider M.R., Mostafa S., Islam S.K. A Low-Power Sensor Read-Out Circuit with FSK Telemetry for Inductively-Powered Implant System.

[68] El-Gabaly A., Jackson B.R., Saavedra C.E. An L-Band Direct Digital QPSK Modulation in CMOS.

[69] Sonkusale S., Luo Z. A Complete Data and Power Telemetry System Utilizing BPSK and LSK Signalling for Biomedical Implants.

[70] Hu Y., Sawan M. A Fully-Integrated Low-Power BPSK Based Wireless Inductive Link for Implantable Medical Devices.

[71] Lu Z., Sawan M. An 8 Mbps Data Rate Transmission by Inductive Link Dedicated to Implantable Devices.

[72] Xu W., Luo W., Sonkusale S. (2009). Fullly Digital BPSK Demodulator and Multi Level LSK Back Telemetry for Biomedical Implant Transceivers. IEEE Trans. Circ. Syst. II.

[73] Lee S.-Y., Cheng C.-J., Liang M.-C. (2011). A Low-Power Bidirectional Telemetry Device with a Near-Field Charging Feature for a Cardiac Microstimulator. IEEE Trans. Biomed. Circ. Syst.

[74] Hu Y., Sawan M. (2005). A Fully Integrated Low-Power BPSK Demodulator for Implantable Medical Devices. IEEE Trans. Circ. Syst.

[75] Deng S., Hu Y., Sawan M. A High Data Rate QPSK Demodulator for Inductively Powered Electronics Implants.

[76] Luo Z., Sonkusale S. (2008). A Novel BPSK Demodulator for Biological Implants. IEEE Trans. Circ. Syst. Part I.

[77] Zhou M., Liu W., Wang G., Sivaprakasam M., Yuce M.R., Weiland J.D., Humayun M.S. A Transcutaneous Data Telemetry System Tolerant to Power Telemetry Interference.

[78] Zhou M., Liu W. A Non-Coherent PSK Receiver with Interference-Canceling for Transcutaneous Neural Implants.

[79] Zhou M., Yuce M.R., Liu W. (2008). A Non-Coherent DPSK Data Receiver with Interference Cancellation for Dual-Band Transcutaneous Telemetries. IEEE J. Sold State Circ.

[80] Gong C.-S.A., Yao K.-W., Shiue M.-T., Lin P.-Y., Huang C.-N. A Miniaturized PSK Demodulation Device fo Short-Range Wireless Neural Prosthetic Systems.

[81] Kim J., Chae M.S., Wu L., Liu W. A Fully Integrated DPSK Demodulator for High Density Biomedical Implants.

[82] Li C.P., Wu Z.H., Li B. A New Integrated Low-Power BPSK Demodulator for Wireless Implantable Neural Recording System.

[83] Sonkusale S., Luo Z. A Wireless Data and Power Telemetry System Using Novel BPSK Demodulator for Non-Destructive Evaluation of Structures.

[84] Xu W., Luo Z., Sonkusale S. Biomedical Implant Transceiver with Novel Multi Level LSK Back Telemetry and Fully Digital BPSK Demodulation.

[85] Elamary G., Chester G., Neasham J. An Analysis of Wireless Inductive Coupling for High Data Rate Biomedical Telemetry Using a New VHDL n-PSK Modulator.

[86] Elamary G., Chester G., Neasham J. A Simple Digital VHDL QPSK Modulator Designed Using CPLD/FPGAs for Biomedical Devices Applications.

[87] Kim D.K., Lee H.S. (2009). Phase-Silence-Shift-Keying for Power-Efficient Modulator. IEICE Trans. Commun.

[88] Asgarian F., Sodagar A.M. A High-Data-Rate Low-Power BPSK Demodulator and Clock Recovery Circuit for Implantable Biomedical Devices.

[89] Asgarian F., Sodagar A.M. A Carrier-Frequency-Independent BPSK Demodulator with 100% Data-Rate-to-Carrier-Frequency Ratio.

[90] Baikoussis N.G., Apostolakis E., Papakonstantinou N.A., Sarantitis I., Dougenis D. (2011). Safety of Magnetic Resonance Imaging in Patients with Implanted Cardiac Prostheses and Metallic Cardiovascular Electronic Devices. Ann. Thorac Surg.

[91] Baikoussis N.G., Karanikolas M., Siminelakis S., Matsagas M., Papadopoulos G. (2010). Baseline Cerebral Oximetry Values in Cardiac and Vascular Surgery Patients: A Prospective Observational Study. J. Cardiothorac. Surg.

[92] He X. (2004). Fully Integrated Transceiver Design in SOI Process.

[93] Maymandi-Nejad M., Sachdev M. A Novel Auto-Zero Technique for a Bio-Implantable Blood Pressure Monitoring Device.

[94] Ershadi M.H., Poudeh M.B., Eshtehardiha S. Fuzzy Logic Controller Based Genetic Algorithm on the Step-Down Converter.

